# Evaluating the performance of CHIRPS and CPC precipitation data for streamflow forecasting using multiple linear regression and Long Short-Term Memory Neural Network model

**DOI:** 10.1016/j.mex.2025.103443

**Published:** 2025-06-17

**Authors:** Khairul Hasan, Md Sahidul Islam, Khayrun Nahar Mitu, Mahade Ibn Salam, Nazli Aghashahi

**Affiliations:** a Department of Civil Engineering, University of Memphis, Memphis, TN 38152, USA; b Department of Earth Science, University of Memphis, Memphis, TN 38152, USA; c Department of Civil and Environmental Engineering, Shahjalal University of Science and Technology, Sylhet, Bangladesh

**Keywords:** CHIRPS, CPC, Streamflow forecasting, Long Short-Term Memory Neural Network, Multiple linear regression, Machine learning approach for streamflow forecasting using gauge-based and satellite-based rainfall data

## Abstract

Accurate streamflow forecasting is essential for effective water resources management and planning. Traditionally, streamflow prediction has relied heavily on a large volume of precipitation data from ground-based weather stations, which are often expensive to build and maintain and provide rainfall data at a coarse spatial resolution. However, machine learning techniques have introduced cost-effective tools for streamflow forecasting that require minimal input data. This study evaluates two prediction models utilizing gauge-based (CPC) and satellite-based (CHIRPS) rainfall data for the Wolf River watershed, comparing their effectiveness in streamflow forecasting. Employing Multiple Linear Regression (MLR) and Long Short-Term Memory Neural Network (LSTM-NN) models, daily precipitation data from Climate Hazards Group InfraRed Precipitation with Station (CHIRPS) and NOAA Climate Prediction Center (CPC) spanning the period from 1991 to 2021, were used for streamflow prediction. The models were developed using daily stream flow data from the Wolf River Watershed stream gauge USGS 07031650 during the specified timeframe. Results indicate that CHIRPS data outperforms CPC data when used with the LSTM-NN model, yielding lower Root Mean Square Error (RMSE) and Mean Absolute Error (MAE) values at 15.02 and 21.53, respectively. Therefore, CHIRPS data emerges as a viable alternative rainfall data source for this study area in scenarios where gauge-based data is unavailable.

The main contribution of this study includes:

• Demonstrating CHIRPS as a viable alternative to gauge-based CPC data for streamflow forecasting.
• Showing that LSTM-NN outperforms MLR in streamflow prediction, achieving lower RMSE and MAE values.

## Graphical abstract

**Figure:**
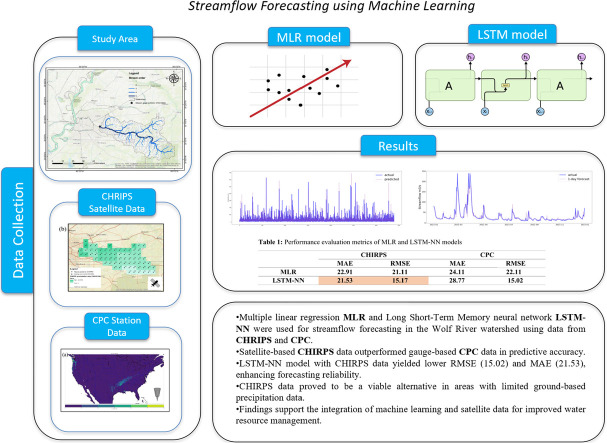

Specifications table

**Table:** 

Subject area:	Engineering
More specific subject area:	Hydrological modeling and machine learning-based streamflow forecasting
Name of your method:	Machine learning approach for streamflow forecasting using gauge-based and satellite-based rainfall data
Name and reference of original method:	Sulugodu, B., & Deka, P. C [1]. Evaluating the performance of CHIRPS satellite rainfall data for streamflow forecasting. Water Resources Management, 33, 3913–3927. https://doi.org/10.1007/s11269-019-02340-6
Resource availability:	None

## Background

Forecasting streamflow provides invaluable insights for effective water resources management, flood mitigation, and the issuance of drought warnings. The design of hydraulic structures and flood forecasts heavily relies on streamflow data [[2], [3], [4]]. However, obtaining such data can be a challenging endeavor, often necessitating intricate field setups and sophisticated measurement techniques. Rainfall-runoff models serve as essential tools for replicating streamflow, yet the prediction of streamflow remains a challenging task due to the multitude of factors influencing runoff, including rainfall patterns and catchment properties [[5], [6], [7]]. The rainfall-runoff process itself is complex and nonlinear, meaning the relationship between rainfall and runoff is not proportional. Small changes in rainfall can induce larger runoff responses, especially if the catchment is saturated. Runoff also depends on prior rainfall events creating a time-varying and cumulative response. This complexity presents key challenges for forecasting as nonlinear processes are difficult to parameterize and predictions are highly sensitive to the accuracy and resolution of various input data, such as precipitation, evapotranspiration, vegetation, soil type, land use, slope, overland flow, infiltration, groundwater level, and more [8].

Meanwhile, process-based hydrological models [9,10], while offering a comprehensive approach, require intricate geological setups and a wealth of observation data. Consequently, the hydrology and water resource management field continue to grapple with the development of a reliable and robust streamflow model. This persistent challenge underscores the critical importance of ongoing attention and exploration in this vital area of study.

While physical models remain valuable for conceptualizing hydrologic phenomena and estimating catchment yields, Machine Learning (ML) models have demonstrated a superior ability to accurately predict essential hydrological parameters due to their ability to learn complex patterns and relationships from the data without explicit hydrological modeling [[11], [12], [13], [14], [15]]. Machine learning models can offer a cost-effective means of simulating streamflow based on limited meteorological inputs, particularly precipitation [[16], [17], [18],^15^]. However, the effectiveness of these models typically hinges on the availability of a substantial amount of data to establish robust relationships between input variables and output predictions, circumventing the need for explicit knowledge of the physical processes involved. The World Meteorological Organization (WMO) stipulates that a minimum of 30 years of long-term precipitation data is essential for conducting climatological research, such as streamflow forecasting [19]. Despite this requirement, operational precipitation products are often characterized by either low-resolution with long-term estimates or high-resolution with short-term estimates [19].

The CHIRPS satellite, designed for rainfall measurement, represents a relatively recent innovation that has not yet undergone comprehensive global validation. This study aims to evaluate the reliability of the CHIRPS satellite in forecasting streamflow, by comparing it with the CPC unified gauge gridded data. The study employs CHIRPS and CPC daily precipitation data spanning the period from 1991 to 2021, utilizing Multiple Linear Regression (MLR) and Long Short-Term Memory Neural Network (LSTM-NN) models to forecast streamflow. The streamflow data selected for model validation originates from the USGS stream gauge 07031650, located at the outlet of the Wolf River Watershed. Statistical measures are employed to rigorously evaluate the effectiveness of the MLR and LSTM-NN models in predicting streamflow.

## Method details

**Fig. 1:**
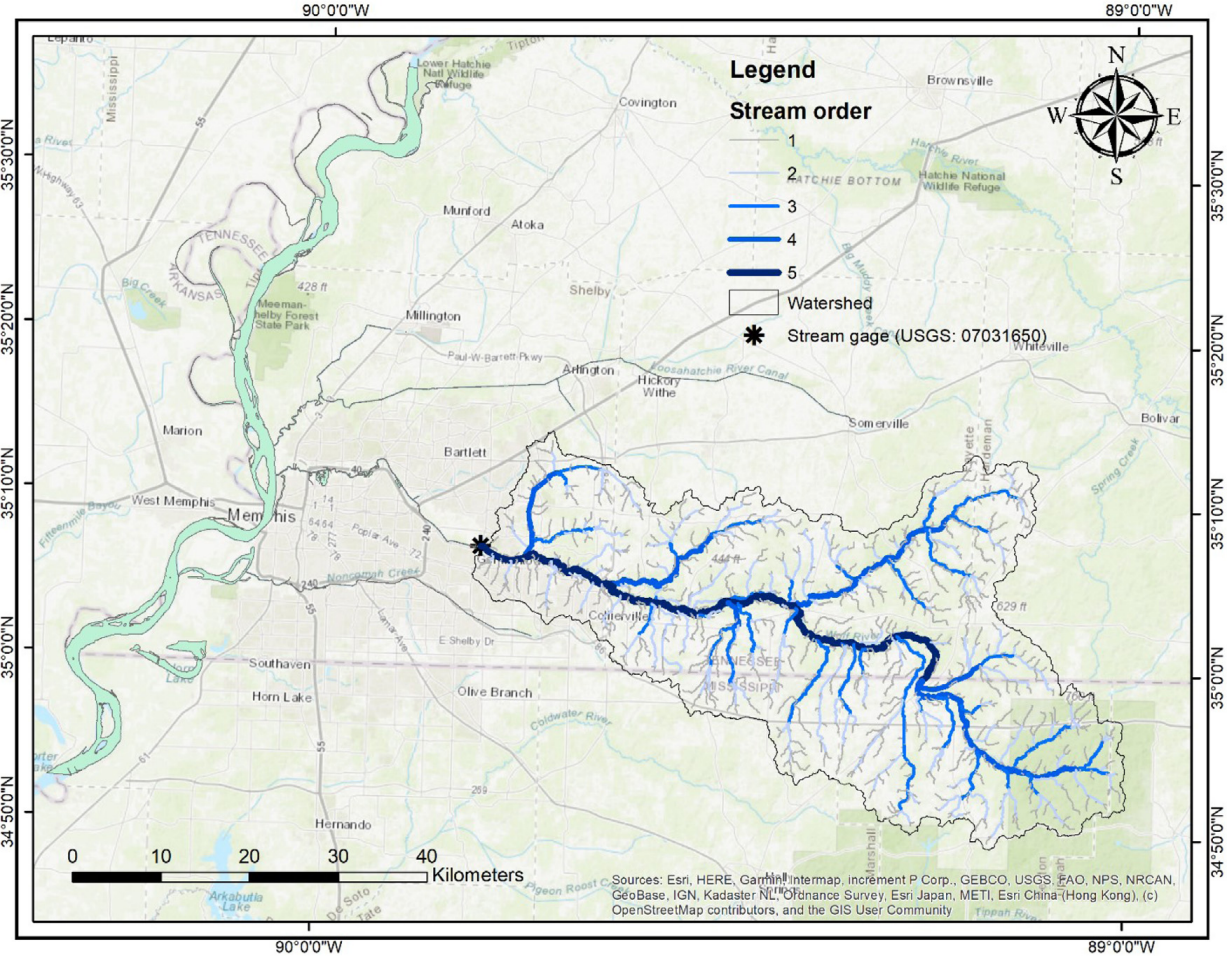
The Wolf River watershed delineated based on the stream gage USGS 07,031,650 [20].

The study has been carried out within the Wolf River watershed, covering a total area of 1805.74 sq. km [20]. The floodplain of this watershed comprises Holocene, saturated, unconsolidated sand overlain by clayey silt [21]. Situated in a humid-temperate climate, the Wolf River watershed experiences a distinct rainy season from October to March, followed by a dry season from April to September. The delineation of the watershed was based on the stream gauge location (USGS: 07031650) situated at Latitude 35°06′59″ and Longitude 89°48′05″. This stream gauge, located at Wolf River in Germantown, TN, serves as the outlet for the study area. Fig. 1 illustrates the delineated watershed, highlighting various stream orders within the region.

The streamflow data for the Germantown Station (USGS 07031650) was obtained from the United States Geological Survey (USGS) website. A comprehensive analysis and model development were conducted utilizing the daily streamflow data from 1 January 1991 to 31 December 2021.

Precipitation data in NetCDF format were sourced from the CHIRPS website. Given the substantial memory requirements for processing and visualizing the global CHIRPS dataset, which has a spatial resolution of 0.05° latitude by 0.05° longitude, the data underwent selective reduction. Specifically, the dataset was constrained to a bounding box encompassing the contiguous United States, with latitudes ranging from 24.0°N to 50.0°N and longitudes from −125.0°W to −66.5°W. Although the primary focus of this study is the Wolf River watershed in Tennessee, this broader bounding box was employed to ensure complete spatial coverage of the watershed and to facilitate potential edge-buffering during spatial analyses. This method significantly reduced the data volume while retaining all pertinent precipitation information for the study area.

**Fig. 2:**
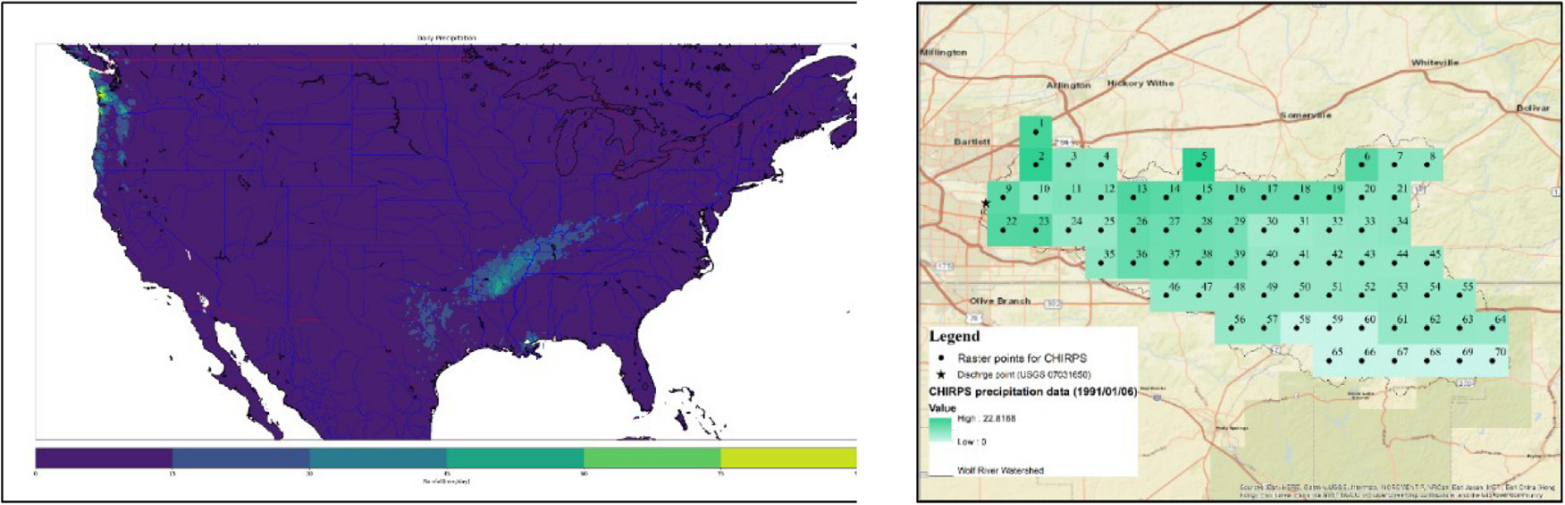
(a) CHIRPS data for the Contiguous USA on 1/1/1991 (b) CHIRPS raster point that covers the Wolf River watershed.

Fig. 2(a) depicts a subset of CHIRPS precipitation data for the Contiguous United States on January 1st, 1991. Thus, the daily time series data from January 1st, 1991 to December 31st, 2021 were extracted. This was done by identifying the raster points within the wolf river watershed, as illustrated in Fig. 2(b).

**Fig. 3:**
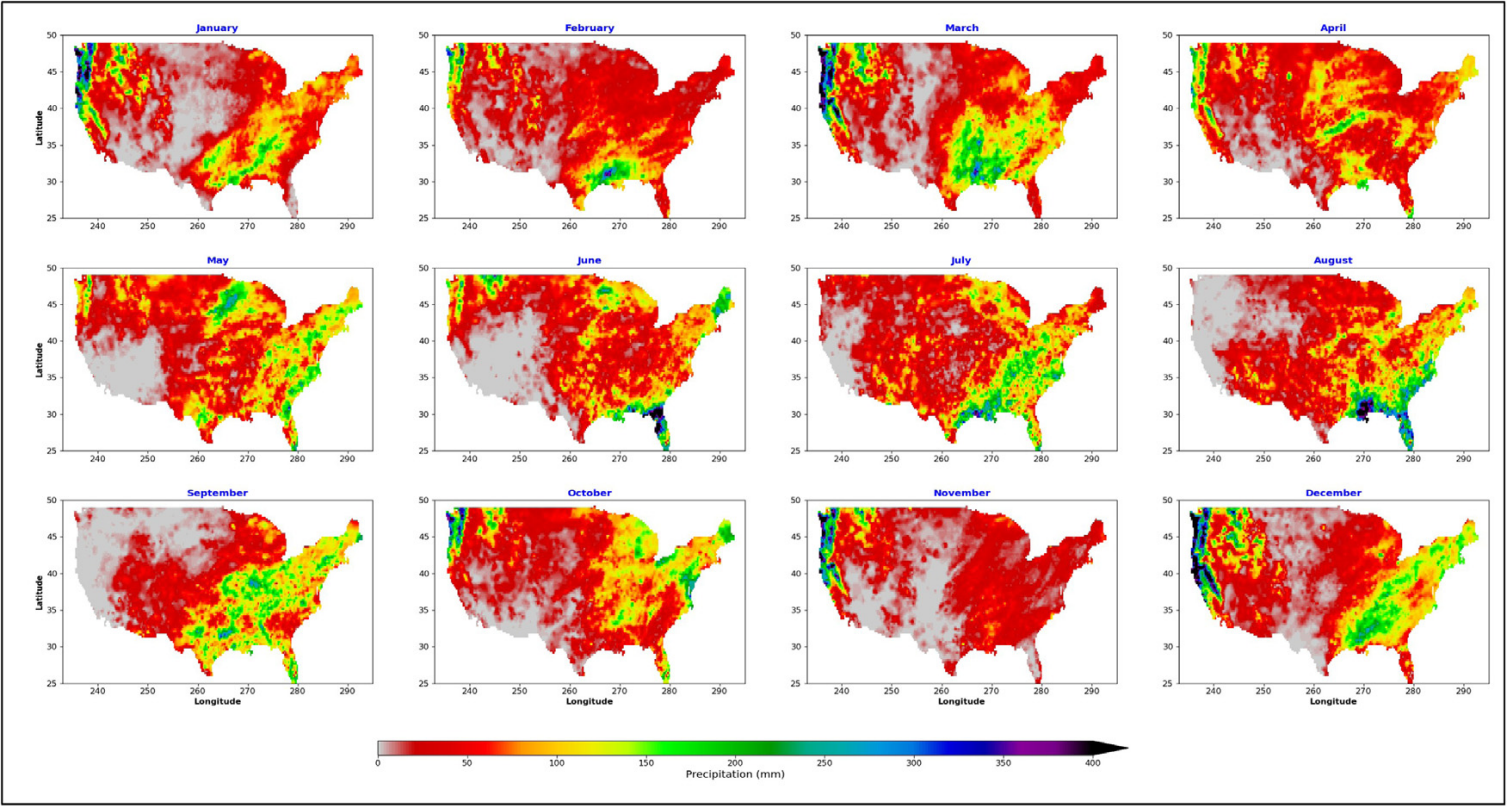
Monthly sum of CPC Unified Gauge-Based daily precipitation for the year 2012.

Similarly, the CPC Unified Gauge-Based daily precipitation over CONUS provided by the NOAA PSL, Boulder, Colorado, United States was obtained. Fig. 3 displays the monthly total precipitation for the entire United States. Then, NetCDF time series data were extracted for multiple geographic coordinates for the same raster point as CHIRPS data.

### Estimation of areal precipitation

Precipitation over a designated catchment area can be characterized through a dense network of point measurements derived from satellite observations rather than relying solely on limited individual point measurements, like those obtained from rain gauges. However, to comprehend the impact of this network of point measurements on discharge at the watershed outlet, it is imperative to translate precipitation data into areal estimates [22]. In pursuit of this objective, the arithmetic mean method was used to average all the points that cover the entire watershed, as shown in Fig. 2(b).

### Multiple linear regression (MLR)

The multiple linear regression (MLR) model is used to evaluate the influence of various predictor variables on a response variable [23]. While MLR is widely applied to assess how meteorological factors affect hydrological responses [24,25], its specific application to time-lagged precipitation and streamflow relationships within this watershed context remains underexplored. This study addresses that gap by using MLR to analyze precipitation data across different time lags as independent variables, thereby capturing both immediate and delayed hydrological responses.

MLR was selected for this study due to its simplicity, which offered several key advantages: (1) it enabled clear interpretation of the contribution of each lagged predictor variable; (2) it is computationally efficient and suitable for data-limited or operational settings; and (3) it served as a robust baseline for comparison with more advanced models such as LSTM.

In the context of streamflow forecasting at the outlet, this method considers various factors to achieve a more comprehensive estimate of streamflow prediction. Antecedent flow conditions such as baseflow in the Wolf River were considered. Similarly, antecedent rainfall data and wet/dry conditions can impact the overall accuracy of the MLR model. For this reason, rainfall, and discharge at various time lags (i.e., P(t), P(t-1), P(t-2), P(t-3), P(t-4), P(t-5), P(t-6), P(t-7), Q(t-1)) were considered as independent variables, with present-day and streamflow Q(t) as the dependent variable. Additionally, seven dummy variables, namely dummy P(t-1) to P(t-7) were created. Here, P(t-1) represents rainfall (in inches) one day ago, and dummy P(t-1) = 1 if rainfall occurred one day ago or 0 if no rainfall occurred. Stepwise multiple linear regression was then performed to obtain the best-fit model with the optimal set of independent variables. The selection process was based on minimizing the Akaike Information Criterion (AIC), which balances model fitness and complexity.

### Long short-term memory neural network (LSTM-NN)

**Fig. 4:**
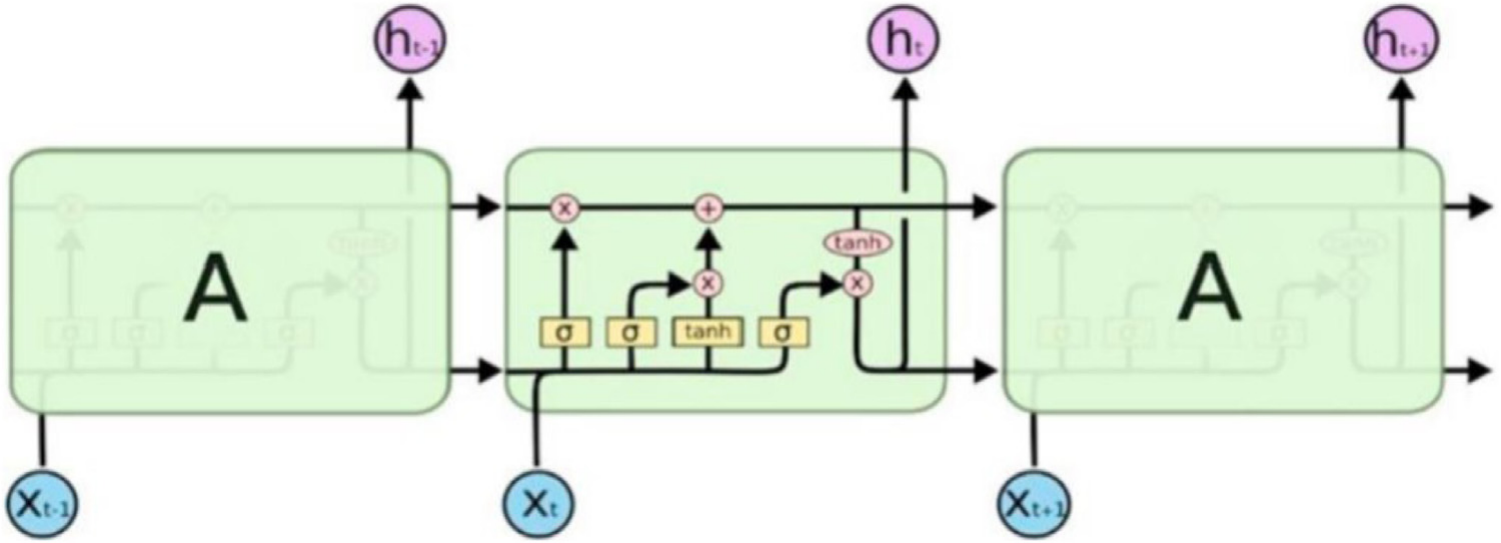
The internal structure of an LSTM [29].

Recurrent Neural Networks (RNNs) are a subclass of neural networks commonly used to solve sequential data problems [26]. RNNs are especially adept at predicting what will occur "next" in sequential data, such as speech and text recognition, financial analysis, weather forecasting [27]. The Long-Short Term Memory (LSTM) model is an improved version of the RNN model [28]. In this study, the LSTM model was selected over the traditional RNN based on previous research demonstrating its ability to capture long-term dependencies in time series data [29,28]. This characteristic is particularly important for hydrological applications, where streamflow responses often exhibit delayed effects due to processes such as infiltration and baseflow. Fig. 4 illustrates the composition of LSTM nodes.

**Fig. 5:**
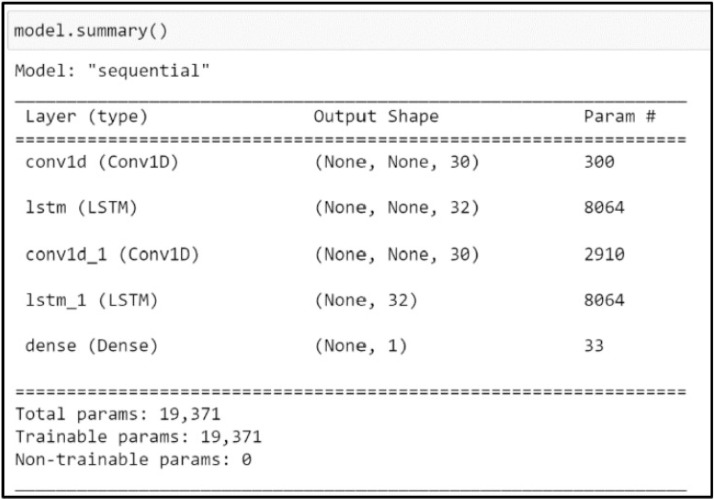
The LSTM-NN model structure used in this investigation.

In the initial step, the structure of LSTM was established. Although the number of layers is subject to judgment, it was found that employing two LSTM layers and one Conv1D layer proved optimal for the dataset used. The optimality of the Conv1D layer depended on several factors, including the temporal resolution of the data, the nature of short-term fluctuations in streamflow, and the model's performance during validation. Specifically, the Conv1D layer helped extract short-term temporal patterns before passing data to the LSTM layers, thereby enhancing the model’s ability to learn both local and long-term dependencies in the streamflow sequence. The number of neurons in each layer is depicted in Fig. 5. The Mean Squared Error (MSE) loss function, and the Adam stochastic gradient descent optimizer were utilized for model optimization. Following the Z-score normalization of the training data, a 3D data structure conforming to the LSTM input format was generated. Subsequently, the data was reshaped into the format (values, timesteps, and 1D output). In this case, the previous 120 days of data were used to predict the next-day streamflow. Therefore, the X-train shape was set as (10,828, 120, 3). The neural network was trained for 1000 epochs with early stopping criteria applied, and a batch size of 32. Once trained, the LSTM model was employed to predict streamflow for the last 365 days of the testing dataset.

### Model performance assessment

Several performance metrics can be used to assess the level of consistency between observed data and predicted streamflow. This study employs a comparative assessment to evaluate the predictability of MLR and LSTM models through graphical representations that contrast actual testing data against predicted testing data. In addition, the performance of these models is quantitatively evaluated using the Root Mean Squared Error (RMSE) and Mean Absolute Error (MAE) [30].

The MAE metric assesses the average magnitude of the model's prediction errors, regardless of whether they are overestimates or underestimates. On the other hand, the RMSE quantifies the difference between observations and predictions in terms of residuals [30]. Since it involves squaring the errors, RMSE is particularly sensitive to larger deviations. Both the MAE and RMSE scores range from zero to infinity. A value of zero for either metric signifies a perfect match between the predicted or observed data, indicating that the model's predictions align precisely with the actual values. As the values of MAE or RMSE increase, they indicate a growing disparity between predicted and observed data, with higher values reflecting greater prediction errors. Therefore, lower MAE and RMSE scores are indicative of more accurate and precise model predictions relative to the observed data [31].

(1) $$MAE=\frac{\sum_{i=1}^{n}\left|Y_{t}\left(obs\right)-Y_{t}\left(pred\right)\right|}{n}$$

(2) $$RMSE=\;\sqrt{\sum_{t=1}^{n}\frac{{\left[Y_{t}\left(obs\right)-Y_{t}\left(pred\right)\right]}^{2}}{n}}$$

## Method validation

### Multiple linear regression (MLR)

**Fig. 6:**
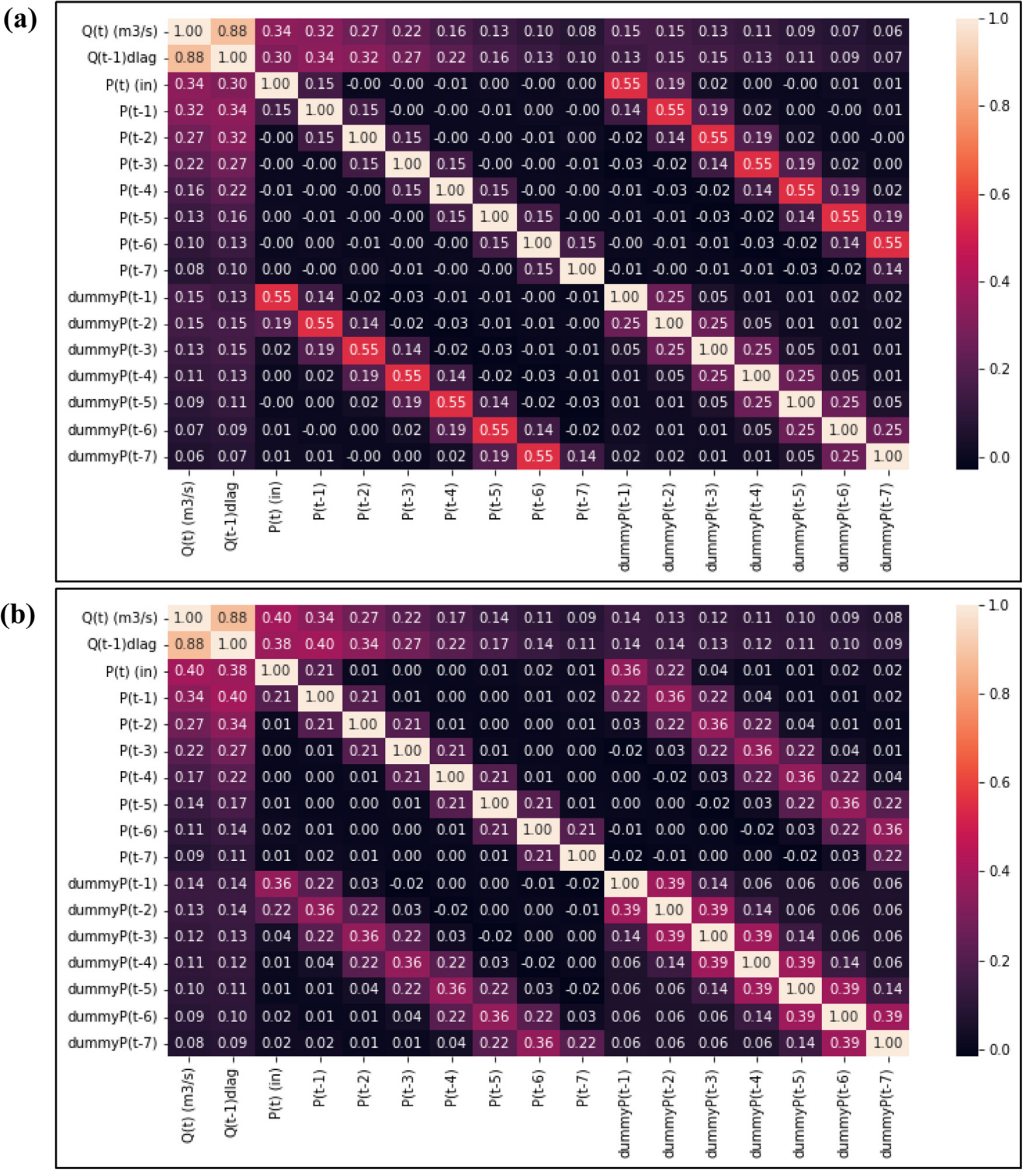
Multi-collinearity test (a) CHIRPS (b) CPC data.

The results of the multi-collinearity test, as depicted in Fig. 6, affirm the absence of collinearity among the variables used in model development. The dataset was partitioned into training (70%) and testing (30%) subsets. Following the stepwise regression process, the final model characterized by optimal parameters, is expressed as follows:

[CHIPRS data]: Q(t) = 2.74 + 0.90Q(t-1) + 6.38P(t) + 2.9P(t-1) - 4.4P(t-3) - 2.45P(t-4) - 2.55P(t-5) – 1.31 dummy P(t-2) +1.52 dummy P(t-4)

[CPC data]: Q(t) = 2.26 + 0.89Q(t-1) + 0.3P(t) −0.16P(t-2) – 0.11P(t-3) – 0.1P(t-4) – 0.05P(t-6) – 0.03P(t-7) −0.92dummyP(t-1) + 1.07dummyP(t-3) + 1.4 dummy P(t-4) + 1.13 dummy P(t-5)

**Fig. 7:**
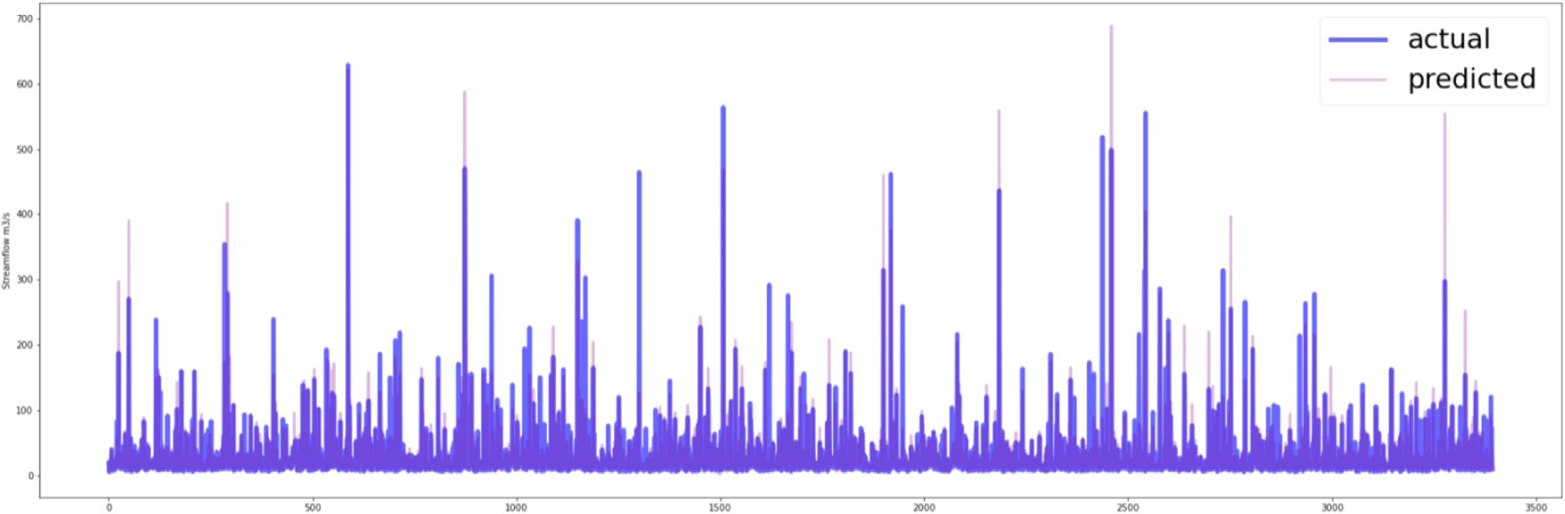
Actual vs predicted plot of testing data (CHIRPS data).

**Fig. 8:**
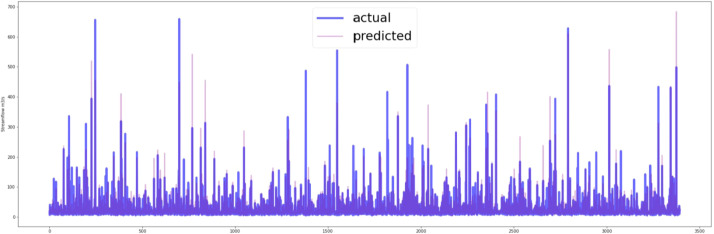
Actual vs predicted plot of testing data (CPC data).

Fig. 7, Fig. 8 show the predictive capability of the MLR model with both CHIRPS and CPC data. The visual representation clearly indicates that the MLR model using CHIRPS data provides a superior fit to the testing dataset.

### LSTM-NN model

**Fig. 9:**
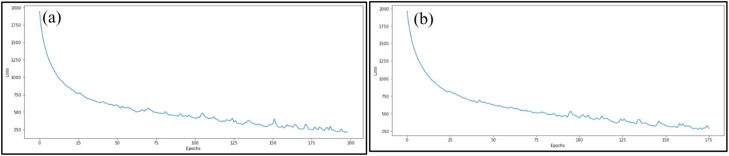
Loss with the number of Epochs (a) CHIRPS (b) CPC.

Fig. 9 depicts the loss function against the number of epochs for both datasets. The graph illustrates a decline in the loss functions over epochs until the Early Stopping criteria are met.

**Fig. 10:**
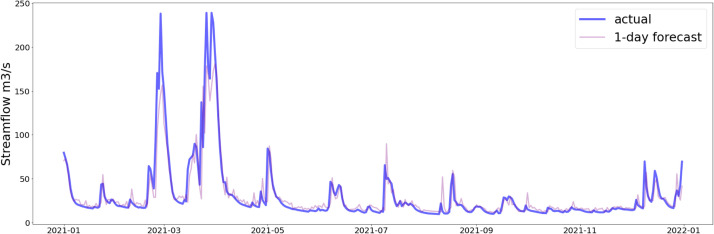
Prediction on the testing dataset of last 365 days (CHIRPS).

**Fig. 11:**
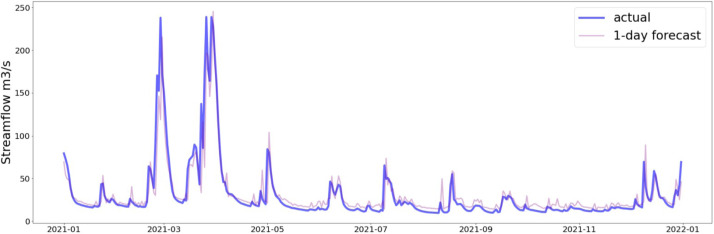
Prediction on the testing dataset of the last 365 days (CPC).

In Fig. 10, Fig. 11, the predictive capability of the LSTM-NN model with both CHIRPS and CPC data is demonstrated. The model exhibits a notable ability to capture the underlying trends and variability within the time series data. The 1-day forecast of the LSTM-NN model using CHIRPS data visually aligns more closely with the observed data.

Table 1 shows that CHIRPS data produce comparable results to CPC. LSTM-NN model using CHIRPS data demonstrates better predictive performance, with lower MAE and RMSE values compared to the model trained with CPC data.

**Table 1:** Performance evaluation metrics of MLR and LSTM-NN models.

	CHIRPS		CPC	
	MAE	RMSE	MAE	RMSE
MLR	22.91	21.11	24.23	22.11
LSTM-NN	21.53	15.02	28.77	15.62

## Summary and discussion

This study highlights the significance of leveraging machine learning models, specifically Multiple Linear Regression (MLR) and Long Short-Term Memory Neural Networks (LSTM-NN), in the realm of streamflow forecasting within the Wolf River watershed. The comprehensive assessment of gauge-based (CPC) and satellite-based (CHIRPS) rainfall data underscores the substantial impact of precipitation data source selection on the predictive accuracy of the models.

The MLR model, carefully tailored with optimal parameters derived from the stepwise regression process, revealed distinct patterns in its equation formulation for both CHIRPS and CPC datasets. Notably, the MLR model with CHIRPS data demonstrated superior performance on the testing dataset, affirming the efficacy of satellite-based rainfall data in enhancing streamflow predictions.

The LSTM-NN model, designed to capture the intricate temporal dynamics of streamflow, exhibited a remarkable ability to discern underlying trends and variability in the time series. The study also portrayed the model's effectiveness in providing a 1-day forecast using CHIRPS data, showcasing a visual fit that surpassed predictions based on CPC data.

Furthermore, the quantitative assessment, as presented in Table 1, conclusively indicates that CHIRPS data yields comparable or even superior results when compared to CPC data. The LSTM-NN model, when fed with CHIRPS data, outperforms its counterpart trained with CPC data, as evidenced by lower Mean Absolute Error (MAE) and Root Mean Square Error (RMSE) values.

This study demonstrates that CHIRPS data, when integrated with machine learning models, can serve as a robust input for streamflow forecasting in data-limited regions. The strong performance of the LSTM-NN model confirms that the potential of deep learning methods for capturing temporal variability in streamflow while MLR offers a transparent baseline for comparison. These findings support continued exploration of satellite-based inputs and machine learning frameworks in hydrological applications.

## Limitations

This research not only solidifies the effectiveness of machine learning techniques but also highlights the practical utility of satellite-based precipitation data, specifically CHIRPS, as a reliable alternative in scenarios where gauge-based data is unavailable. The emphasis on the potential application of CHIRPS data holds particular importance for water resource management, offering a viable solution to enhance forecasting accuracy in regions with limited ground-based monitoring infrastructure.

Future research could expand by exploring a broader range of machine learning algorithms, including ensemble methods (e.g., Random Forest, Gradient Boosting), advanced deep learning models (e.g., Convolutional Neural Networks, Transformer architectures), or hybrid approaches that combine physical and data-driven models. Additionally, future studies could refine model architecture, experiment with alternative input features, and extend the evaluation of CHIRPS data across diverse climatic and hydrological contexts.

Further investigation is also warranted into the uncertainty associated with satellite-derived precipitation products, especially under extreme rainfall conditions or in regions with complex terrain. Quantifying and reducing these uncertainties will be essential for operational applications.

Expanding forecast lead times beyond one day and evaluating model generalization across other watersheds with different hydrologic behaviors would also help validate the broader applicability of the approach.

In summary, this study contributes to the evolving field of streamflow forecasting and underscores the importance of integrating innovative machine learning techniques and satellite-based data to improve water resource prediction accuracy.

## Ethics statements

This study does not involve human subjects, animal experiments, or data collected from social media platforms. All data used in this research, including precipitation and streamflow records, were obtained from publicly available sources and do not contain any personally identifiable information. Therefore, no ethical approval or informed consent was required.

## CRediT author statement

**Khairul Hasan:** Conceptualization, Methodology, Writing – original draft, review & editing. **Md Sahidul Islam:** Methodology, Data analysis, Writing – original draft. **Khayrun Nahar Mitu:** Data analysis, Validation, Writing – review & editing. **Mahade Ibn Salam:** Visualization, Writing – review & editing. **Nazli Aghashahi:** Validation, Writing – review & editing.

## Declaration of competing interest

The authors declare that they have no known competing financial interests or personal relationships that could have appeared to influence the work reported in this paper.

## Data Availability

Data will be made available on request.
